# Mass cytometry reveals complex neutrophil heterogeneity in patients with severe sepsis

**DOI:** 10.1186/s40635-026-00924-2

**Published:** 2026-06-11

**Authors:** Patricia D. A. Lima, Christina Yu, Miranda Hunt, Francois Lamontagne, Neill K. J. Adhikari, John C. Marshall, Charles C. T. Hindmarch, David M. Maslove

**Affiliations:** 1 Queen’s Cardiopulmonary Unit, Queen’s University, Kingston, ON Canada; 2 Department of Critical Care Medicine, Queen’s University, Kingston, ON Canada; 3 Department of Medicine, Université de Sherbroooke, Sherbrooke, QC Canada; 4 Department of Critical Care Medicine, Sunnybrook Health Sciences Centre, Toronto, ON Canada; 5 Interdepartmental Division of Critical Care Medicine, University of Toronto, Toronto, ON Canada; 6 Department of Surgery, University of Toronto, Toronto, ON Canada; 7 Department of Medicine, Queen’s University, Kingston, ON Canada; 8 Department of Biomedical and Molecular Science, Queen’s University, Kingston, ON Canada; 9 Kingston Health Sciences Centre, Davies 2 76 Stuart St., Kingston, ON K7L 2V7 Canada

**Keywords:** Sepsis, Neutrophils, CyTOF, Mass cytometry, Precision medicine, Innate immunity

## Abstract

**Rationale:**

Sepsis is a state of life-threatening organ dysfunction in the setting of infection. It is biologically heterogeneous, as evidenced by whole blood transcriptomic analyses that reveal distinct molecular subtypes based on gene expression. At the cellular level, sepsis is primarily mediated by neutrophils, a leukocyte population increasingly recognized as heterogeneous across several domains. We sought to further characterize neutrophil heterogeneity by identifying neutrophil subsets in critically ill patients with severe sepsis based on multidimensional mass cytometry analysis.

**Methods:**

We generated time series mass cytometry (CyTOF) data from whole blood samples taken from 17 patients with sepsis admitted to an intensive care unit who were enrolled in a randomized controlled trial of high-dose vitamin C. We analyzed these data using unsupervised machine learning techniques to identify distinct neutrophil subtypes. We characterized the resulting subtypes and described their changes over time.

**Results:**

We analyzed approximately 1.5 million cytometry events gated as neutrophils. We identified five clusters that reveal complex heterogeneity across multiple neutrophil markers of maturation and activation including olfactomedin-4 (OLFM4), CD177, glucose transporter 1 (GLUT1), CD16, and lipocalin-2. The two dominant clusters differed primarily in the abundance of OLFM4, a neutrophil granule protein. Between Day 1 and Day 7, there was an increased proportion of neutrophils in the dominant OLFM4-expressing cluster, a difference primarily driven by increased abundance of OLFM4 in the placebo group, but not the vitamin C group.

**Conclusions:**

Our findings point to complex multidimensional heterogeneity among neutrophils in sepsis, thereby extending the current concept of neutrophil heterogeneity with new data from critically ill patients with sepsis derived from mass cytometry. Variation in the temporal differences in cluster proportions between treatment arms may suggest opportunities for precision sepsis treatment.

**Supplementary Information:**

The online version contains supplementary material available at 10.1186/s40635-026-00924-2.

## Background

Sepsis is broadly understood as life-threatening organ dysfunction due to infection, but its underlying biological mechanisms may differ from one individual to the next [1]. This heterogeneity has long been evident clinically in the diversity of presentations, outcomes, and responses to treatment seen among patients with infection. More recently, heterogeneity has also been identified in the biology of sepsis, based on more detailed characterization of the molecular and cellular responses to infection [2]. These findings may be key to understanding how to correlate treatment responses with biological states [3].

At the molecular level, evidence of sepsis heterogeneity comes mostly from whole blood analyses of gene expression [4]. These studies have revealed molecular subtypes of sepsis distinguished by shared mRNA expression patterns. Work is ongoing to translate these transcriptional sepsis subtypes into so-called treatable traits—biologic states that portend a predictable response to a specific therapy [5]. But the extrapolation of biological function from mRNA levels alone may be incomplete. Traditional methods of gene set enrichment in bioinformatics workflows may not account for a number of important biological and statistical considerations [6, 7].

At the cellular level, the host response to infection—a key determinant of sepsis—is initially orchestrated by neutrophils as part of the innate immune response [8]. Heterogeneity has been reported here as well, with different neutrophil subtypes identified across different domains, and using different cytometric and molecular profiling techniques [9–11].

In this study, we investigate neutrophil heterogeneity in severe sepsis through mass cytometry, using a panel of antibodies targeting multiple cellular locations and functions. We build on existing knowledge of neutrophil heterogeneity, combining markers to identify distinct neutrophil clusters defined by multiple parameters, as well as their relative abundance over time.

## Methods

### Study design

We conducted a mass cytometry (CyTOF) analysis of whole blood samples from patients enrolled in the Lessening Organ dysfunction with Vitamin C (LOVIT) trial, an international multi-center randomized controlled trial examining the effects of high-dose intravenous vitamin C in critically ill patients with sepsis (NCT03680274) [12]. This study was approved by the Health Science and Affiliated Teaching Hospitals Research Ethics Board (HSREB) at Queen’s University (DMED-2297-19). Patients or their substitute decision makers provided written informed consent prior to any study procedures. We collected 6 ml of blood in BD^®^ Vacutainer Heparin tubes (Beckman, Dickson and Company, BD, USA) from patients admitted to the intensive care unit of Kingston Health Science Center with sepsis, who required vasopressors for blood pressure support (see Supplemental Methods). Blood was collected on Day 1 (prior to the first dose of study medication), and again on Day 7 for patients still in the ICU at that time. For each mL of blood, 1.4 mL of proteomic stabilizer was added (PROT1, SmartTube, Inc.) and the samples were then stored at −80 °C.

### Mass cytometry

Samples were thawed and processed for CyTOF analysis in a single batch (see Supplemental Methods). We used a CyTOF antibody panel with markers chosen to allow for the identification of neutrophils, as well as the characterization of neutrophil maturation and activation (Table 1 and Supplemental Table 1). We also included sodium ascorbate co-transporters (SVCT) and hexose transporters (GLUT), given their roles in vitamin C uptake by neutrophils. All samples were stained simultaneously to reduce technical discrepancies, and the protocol was validated using blood from three healthy controls prior to use with study samples (see Supplemental Methods). Data were acquired using the Helios (Standard Biotools), a high-performance mass cytometer from the CyTOF (time-of-flight detection) family of instruments located at the Queen’s CardioPulmonary Unit (QCPU), Queen’s University. The Cytobank platform (Beckman Coulter Life Science, IN USA) was used to clean the normalization beads as per Standard Biotools guidelines (technical note #400248 Rv 06), and for gating. The gating strategy for identifying neutrophils is shown in Supplemental Fig. 1. Briefly, we excluded red blood cells and platelets by gating on CD45^+^ cells, and subsequently we excluded cells expressing CD3 (T cells), CD19 (B cells), natural killer cells (CD56^+^CD16^−^ or CD56^+^CD16^+^), and monocytes (CD14^+^MPO^−^). Neutrophils were considered CD45^+^CD3^−^CD19^−^CD56^−^CD16^±^CD14^−^ cells that were positive for myeloperoxidase (MPO^+^).

**Table 1:** Antibody panel used in the CyTOF experiment

Marker	Description
Cell lineage markers	
CD45	Protein tyrosine phosphatase receptor type C—the leukocyte common antigen expressed in haematopoietic cells except by erythrocytes and platelets
CD3	T-cell antigen receptor, and part of the T cell receptor (TCR) complex
CD19	A transmembrane glycoprotein that belongs to the immunoglobulin superfamily and is widely expressed on B cells
CD56	Also known as neural cell adhesion molecule, CD56 is a phenotypic marker of natural killer (NK) cells, but also can be expressed on T cells, dendritic cells and monocytes
CD16	Transmembrane receptor containing Ig-like domains. It exists in two isoforms, CD16a, expressed on NK cells, intermediate and non-classical monocytes and macrophages, and CD16b, expressed on neutrophils
CD14	Glycolipid- anchored membrane glycoprotein expressed on myelomonocyte lineage including monocytes, macrophages and some granulocytes
Myeloperoxidase (MPO)	A peroxidase enzyme stored in the azurophilic granules of neutrophils and released during degranulation
Neutrophil activation markers	
CXCR1 (CD181)	Receptor to interleukin 8, which is a neutrophils chemotactic factor—paralog of CD182
CXCR2 (CD182)	Receptor to interleukin 8, which is a neutrophils chemotactic factor—paralog of CD181
CD15 (Sialyl Lewis X antigen)	Cell surface glycan used as a marker for human myeloid differentiation, constitutively expressed at high levels on granulocytes
CD177	A glycosyl-phosphatidylinositol (GPI)-linked cell surface glycoprotein with a role in neutrophil activation and migration
Olfactomedin-4 (OLFM4)	A matrix glycoprotein of neutrophil-specific granules that has been used to distinguished different subsets of neutrophils
TLR4 (CD284)	An endotoxin receptor that mediates the innate immune response to bacterial lipopolysaccharide (LPS) via activating NFκB, cytokine secretion and inflammatory response
TLR2 (CD282)	A receptor that mediates the innate immune response to bacterial lipoproteins and other microbial cell wall components via activating NFκB, cytokine secretion and inflammatory response
Lactoferrin	Also known as lactotransferrin; it is an iron-binding glycoprotein present in secondary granules in neutrophils, and it is released upon neutrophil activation
Lipocalin-2	A glycoprotein, also known as neutrophil gelatinase-associated lipocalin (NGAL), stored in the azurophilic granules of neutrophils
GM-CSFr (CD116)	Receptor for granulocyte–macrophage colony-stimulating factor (GM-CSF), which stimulates proliferation and differentiation of white blood cells. It is expressed on neutrophils, eosinophils, monocytes and macrophages
Transcription factors	
Hypoxia-inducible factor 1-alpha (HIF-1A)	Transcriptional regulator of the adaptive response to hypoxia
Phospho-NFkB p65 (NFκB)	Phosphorylated form of the nuclear factor-NF-kappa-B p65 subunit (also known as a RELA). This subunit is involved in the NFkB heterodimer formation, nuclear translocation and activation. It activates the transcription of several inflammatory genes
Phospho-Extracellular signal-regulated kinase 1/2 (pERK)	Member of the mitogen-activated protein kinase family. Phosphorylated form of ERK1/2 moves to the nucleus and induces the expression of genes involved in cell proliferation, differentiation and apoptosis
Cleaved-Caspase-3 (cCasp3)	Active form of Caspase 3, a critical molecule that induces apoptosis
Vitamin C transporters	
Glucose transporter 1 (GLUT1; Hexose transporter 1)	A uniporter protein encoded by the *SLC2A1* gene. Responsible for the uptake of glucose and dehydroascorbic acid (DHA; oxidized form of AA)
SVCT1 (Solute carrier family 23 member 1; SLC23A1)	Encoded by *SLC23A1* gene. Mediates electrogenic uptake of vitamin C
SVCT2 (Solute carrier family 23 member 2; SLC23A2)	Encoded by *SLC23A2* gene. Mediates electrogenic uptake of vitamin C
Glucose transporter 3 (GLUT3; solute carrier family 2 member 3)	Encoded by *SLC2A3* gene. Facilitates glucose transport but also mediates the uptake of various other monosaccharides across the cell membrane

### Cluster analysis

Cluster analysis of CyTOF data was conducted using the CATALYST package (v.1.34.1) in R [13]. We used dimensionality reduction and unsupervised clustering techniques to visualize and identify distinct neutrophil clusters, based on the abundance of markers, excluding those used for negative gating of neutrophils (CD3, CD14, CD19, CD56). To capture cells in various stages of maturation and activation, we concatenated samples from all patients and time points. We used t-distributed stochastic neighbor embedding (t-SNE) for visualization, and flowSOM for clustering [14]. The optimal number of clusters was determined by identifying the elbow in the area under the cumulative distribution function (CDF) curve. We characterized neutrophil clusters by examining the abundance of the markers and stratified the subsets according to sample time points and individual patients.

### Time series analysis

We examined the differences between Day 1 and Day 7 in the subset of patients with samples from both time points using paired t-tests. We also conducted an exploratory analysis of the potential treatment effects of high-dose vitamin C by comparing the change from Day 1 to Day 7 for patients who were randomized to receive vitamin C, with those who received placebo. We used linear models with empirical Bayesian moderation (limma package) to test for differential abundance between time points and to test for an interaction between treatment arm and time point.

## Results

Between August 2020 and January 2021, we enrolled a convenience sample of 17 patients who were enrolled in the LOVIT trial at our site. Baseline patient characteristics are shown in Table 2. The average patient age was 61.2 ± 10.6 years (10 males and 7 females). Infections were predominantly of the skin and soft tissues (41%) or intra-abdominal (35%). Six patients died during the study, including three who died prior to the second sampling time point. Two patients were discharged from the ICU prior to the second time point. A total of 29 samples (17 from Day 1, 12 from Day 7) were further analyzed.

**Table 2:** Baseline patient characteristics

Characteristic	Patients (*n* = 17)
Age—year	61.2 ± 10.6
Female sex—no. (%)	7 (41)
Comorbidities—no. (%)	
Angina, previous MI	1 (6)
Congestive heart failure	5 (29)
Hypertension	9 (53)
Diabetes	4 (24)
Dialysis prior to admission	2 (12)
Immunosuppression	2 (12)
Admission type—no. (%)	
Medical	10 (59)
Emergency surgery	7 (41)
APACHE II score*	19.9 ± 7.3
Score on clinical frailty scale	
1 to 4—no. (%)	13 (76)
≥ 5—no. (%)	4 (24)
Primary site of infection—no. (%)	
Pulmonary	1 (6)
Gastrointestinal or intra-abdominal	6 (35)
Blood	1 (6)
Skin or soft tissue	7 (41)
Urinary	1 (6)
Other	1 (6)
Mean arterial pressure (mmHg)*	70.4 ± 7
Vasopressors—no. (%)*	
Norepinephrine	17 (100)
Vasopressin	15 (88)
Epinephrine	8 (47)
Corticosteroids given prior to enrollment—no. (%)	11 (65)
Outcomes—no. (%)	
Died in ICU	5 (30)
Died by Day 28	6 (35)
Death or persistent organ dysfunction at Day 28†	10 (59)

Plus–minus values are means ± SD

^*^Denotes value at the time of enrollment

^†^Persistent organ dysfunction defined as ongoing need for new dialysis, vasopressors, or mechanical ventilation

Flow cytometry analysis of the concatenated samples revealed a total of 1,475,508 events gated as neutrophils. Neutrophils were generally the most abundant leukocyte type in the samples. The total white blood cell count (WBC) at Day 1 ranged from 0.6 × 10e9/L to 51.9 × 10e9/L.

We used non-redundancy scores (NRS) to evaluate the contribution of the various markers to the variance in the dataset (Supplementary Fig. 2). Most of the variance was accounted for by differences in OLFM4, CD177, and GLUT1, with lesser contributions from CD16, lipocalin-2, SVCT1, and lactoferrin. Transcription factors contributed comparatively little to the variance.

### Cluster analysis

**Fig. 1:**
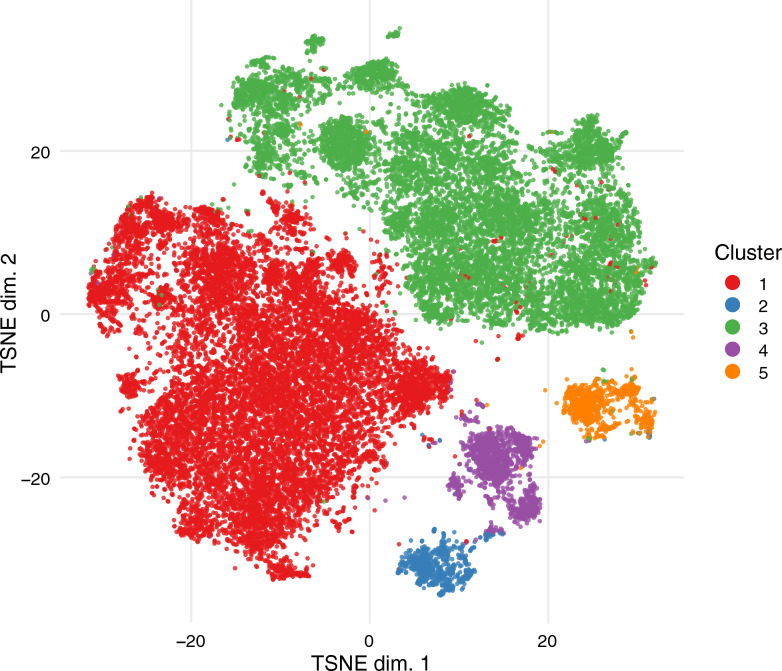
t-SNE plot showing neutrophils clustered according to similarities in the abundance of the various CyTOF markers. The colors in the plot represent the cluster assignment, as determined by the flowSOM clustering algorithm with *k* = 5

**Fig. 2:**
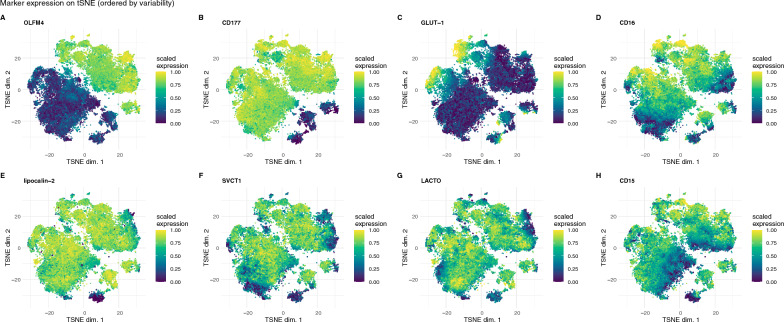
Each panel shows a t-SNE plot in which neutrophils are clustered according to similarities in the abundance of maturation and activation markers. **A** OLFM4; **B** CD177; **C** GLUT1; **D** CD16; **E** lipocalin-2; **F** SVCT1; **G** lactoferrin; **H** CD15

We tested various clustering solutions based on the abundance of the 20 neutrophil-specific markers in the CyTOF panel. Clustering was done using the flowSOM algorithm with a maximum of 20 clusters. Examination of the area under the cumulative distribution function curve suggested that a 5-cluster model was well supported by the data (Supplementary Fig. 2). This model is shown in Fig. 1, with the abundance of the most determinative markers shown in Fig. 2. One small cluster (Cluster 2) was relatively homogeneous, characterized by low abundance of OLFM4, CD177, lipocalin-2, and CD16, suggesting that these cells were immature neutrophils. Most samples contained very few of these Cluster 2 neutrophils (mean 3.6%). The largest clusters were Cluster 1 and Cluster 3, which accounted on average for 49% and 39%, respectively, of the neutrophils in each sample. Both clusters showed high abundance of CD177 (Fig. 2B) and lipocalin-2 (Fig. 2E) but differed in terms of OLFM4 (Fig. 2A). These larger clusters exhibited residual heterogeneity with respect to additional markers including GLUT1 (Fig. 2C), CD16 (Fig. 2D), and SVCT1 (Fig. 2F). Clusters 4 and 5 were also small, accounting on average for 5% and 4%, respectively, of the neutrophils in each sample. The clustered expression levels of the remaining markers are shown in Supplemental Figs. 3 and 4.

**Fig. 3:**
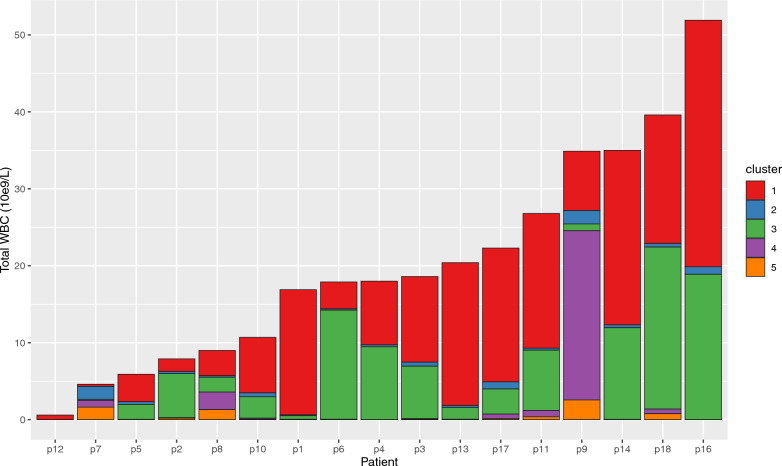
Proportions of neutrophil clusters and Day 1 white blood cell count (WBC). The height of each bar corresponds to the highest WBC on Day 1 for each patient. The coloring represents the proportion of each neutrophil cluster within the total WBC

Consistent with the pooled results, a per-patient analysis showed that most samples at baseline were composed primarily of Clusters 1 and 3 neutrophils, irrespective of the total WBC count in the sample (Fig. 3). Nonetheless, there was some heterogeneity between patients. One baseline sample from a patient with low baseline WBC (p7) showed predominance of Clusters 2, 4, and 5, while another patient (p9) showed predominance of Cluster 4 neutrophils, which were rare in most samples.

### Time series analysis

**Fig. 4:**
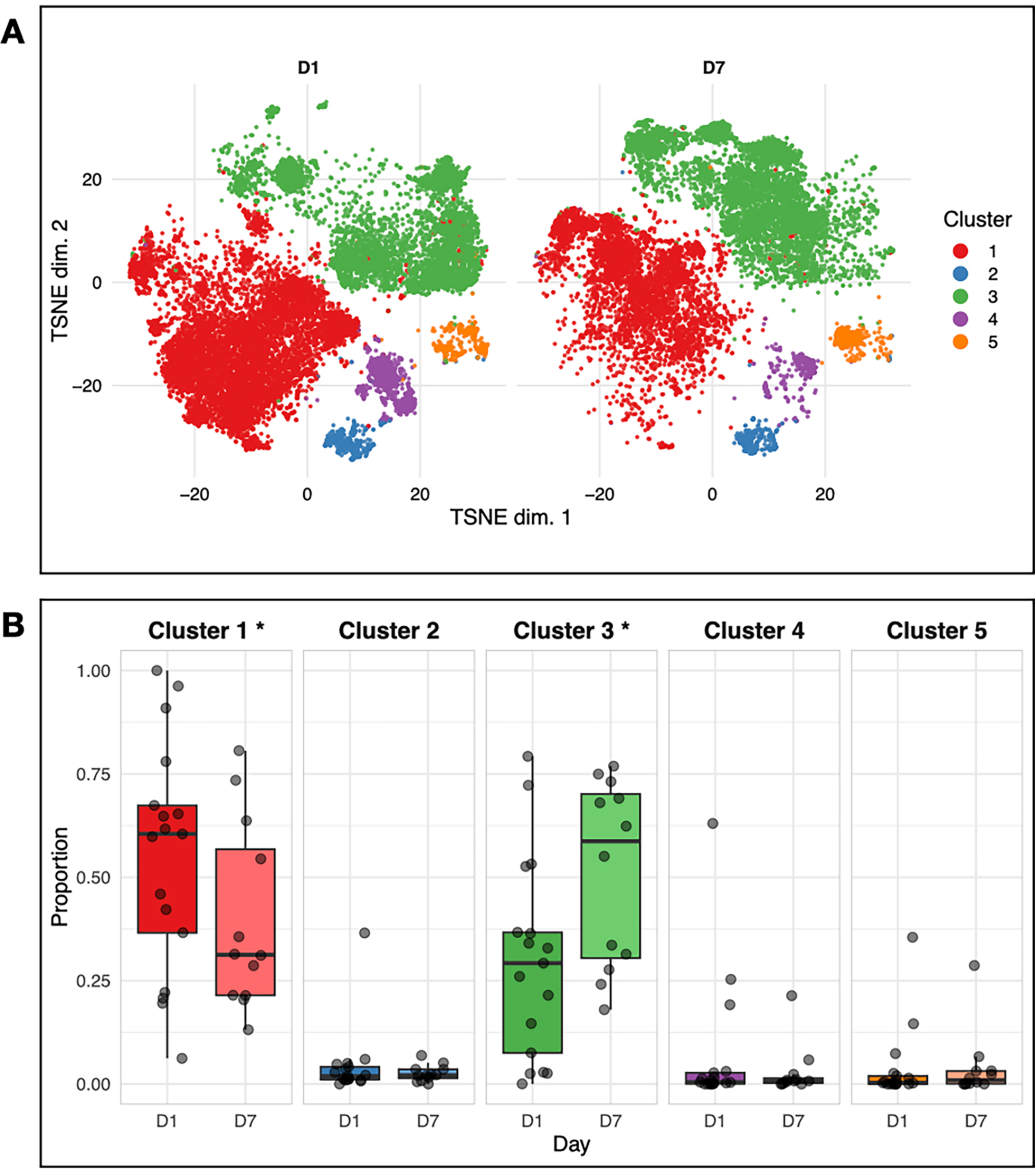
Neutrophil cluster proportions on Day 1 and Day 7. **A** t-SNE plot showing neutrophils clustered according to similarities in the abundance of the CyTOF markers, stratified by day of sampling (left—Day 1, right—Day 7). **B** Paired boxplots indicating the proportion of neutrophils in each cluster at each of the time points. Asterisks indicate significant differences between Day 1 and Day 7 (*p* < 0.05 by paired t-test)

To explore the changes in cluster proportions over time, we conducted a paired analysis of Day 1 and Day 7 samples among patients for whom both were available (Fig. 4). This restricted cohort contained 10% fewer neutrophils overall and 30% fewer patients. Between Day 1 and Day 7 there was an increase in the proportion of Cluster 3 neutrophils (median 35% vs 59%, Cohen’s d 0.601 [0.026–1.177], *p* = 0.04), and a decrease in Cluster 1 neutrophils (median 53% vs 31%, Cohen’s d −0.461 [−0.879 to −0.042], *p* = 0.04).

**Fig. 5:**
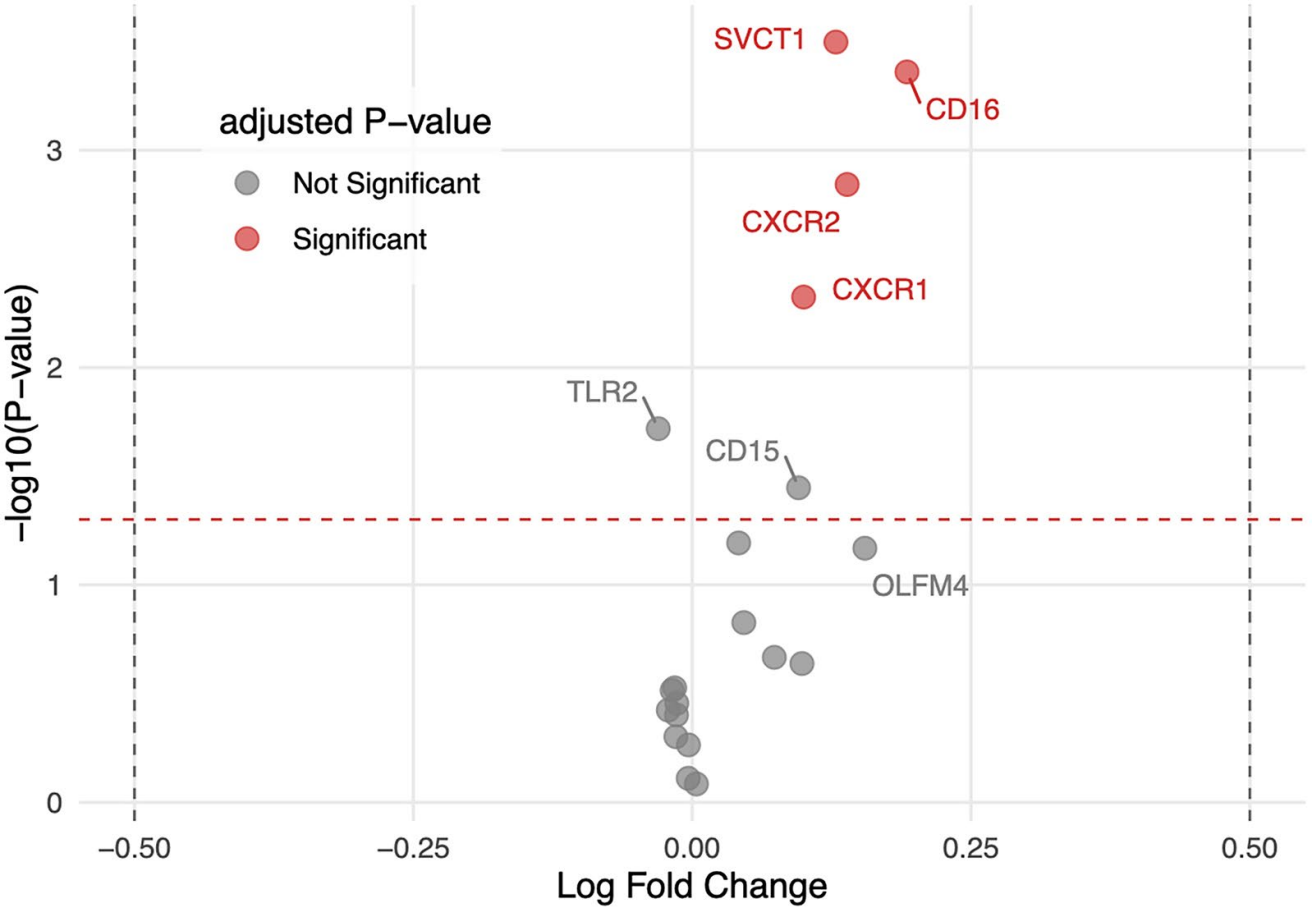
Volcano plot showing differences between Day 1 and Day 7 for individual markers in the CyTOF experiment. Markers colored red were significantly different between time points (Benjamini–Hochberg-adjusted *p* < 0.05). The horizontal dashed line presents an uncorrected *p* < 0.05

Analysis of individual markers revealed slight increases from Day 1 to Day 7 in the abundance of SVCT1, CD16, CXCR2, CXCR1, and CD15, as well as a slight decrease in TLR2 (Fig. 5). These latter two changes were no longer statistically significant after correction for multiple comparisons (Benjamini-Hochberg).

**Fig. 6:**
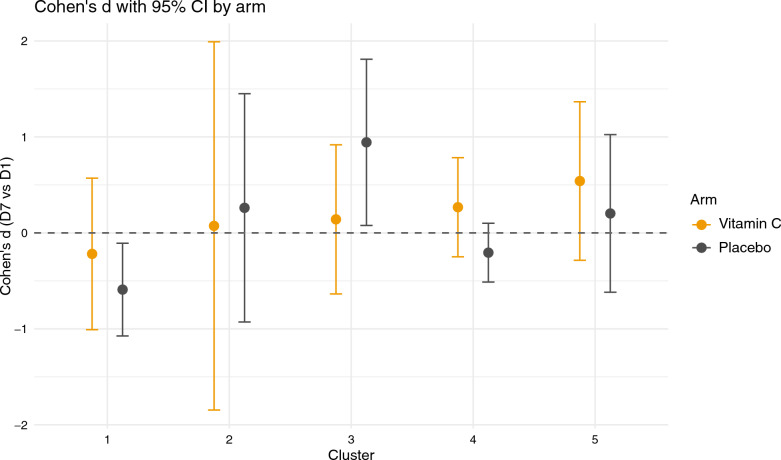
Exploration of the effects of vitamin C on cluster composition. Each set of points with error bars shows the difference in cluster proportion between Day 1 and Day 7 shown as Cohen’s d values. Results are stratified by treatment arm. In the placebo arm, there was a significant decrease in the proportion of Cluster 1, as well as a significant increase in the proportion of Cluster 3. None of the changes between Day 1 and Day 7 was significant in the placebo arm

To explore potential treatment effects of vitamin C, we compared the changes in cluster proportions over time, stratified by treatment arms (Fig. 6). In the placebo arm, there was a substantial increase in the mean proportion of Cluster 3 neutrophils (median 34% vs 55%, Cohen’s d 0.944 [0.078–1.81], *p* = 0.03), alongside a decrease in Cluster 1 neutrophils (median 46% to 31%, Cohen’s d −0.591 [−1.07 to −0.11], *p* = 0.03). There were no differences between Day 1 and Day 7 in the vitamin C arm.

**Fig. 7:**
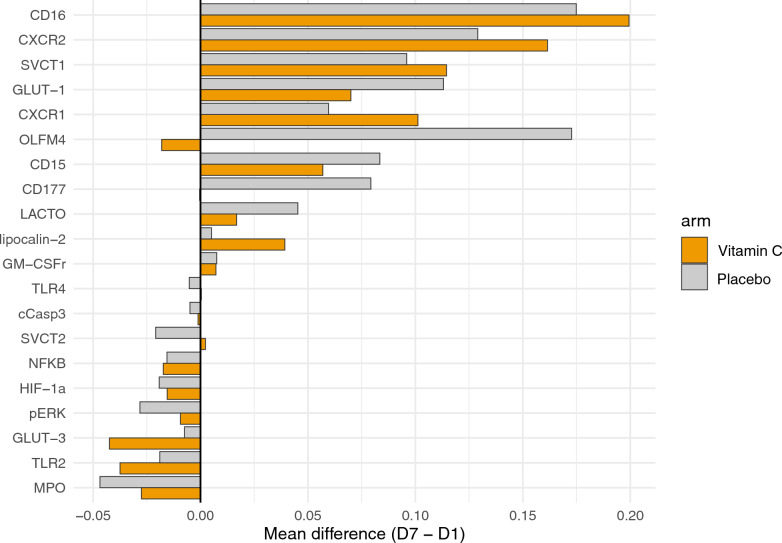
Exploration of the effects of vitamin C on individual marker expression. Each pair of bars shows the relative change in abundance of each marker, stratified by treatment arm. The directionality of change from Day 1 to Day 7 was discordant between the two arms for OLFM4, CD177, TLR4, and SCVT2

We also evaluated the changes in the individual markers stratified by treatment arm (Supplemental Table 2). Prior to adjustment of *p*-values for multiple comparisons, there was an increase in the mean SVCT1 expression in both arms (placebo + 20%, vitamin C + 28%), and a slight decrease in the mean MPO expression in both arms (placebo −5%, vitamin C −3%). There was an increase in OLFM4 in the placebo arm (+ 37%), but not in the vitamin C arm. There was an increase in CXCR1 in the vitamin C arm (+ 41%), but not in the placebo arm. The expression of pERK decreased in the placebo arm only (−17%). These differences were no longer significant after Benjamini–Hochberg correction for multiple hypothesis testing. The directionality of change from Day 1 to Day 7 was discordant between the two arms for OLFM4, CD177, TLR4, and SCVT2 (Fig. 7). Changes between Day 1 and Day 7 for all markers are shown in Supplementary Fig. 5.

To further test for differences between treatment arms we used linear modeling with Bayesian moderation (limma package) to look for an interaction between study arm (vitamin C vs placebo) and study day (Day 1 vs Day 7). We found no statistically significant interaction between study arm and study day for any of the clusters or markers.

## Discussion

In this study, we used mass cytometry to identify subsets of neutrophils distinguished by the relative abundance of neutrophil differentiation and activation markers. With this technique, we were able to evaluate markers at the protein level in individual neutrophils, enabling the discovery and quantification of specific neutrophil subsets. We strategically concatenated the samples to enable the detection of canonical clusters and evaluated samples from individual patients and time points. Our study contributes to the growing characterization of neutrophil heterogeneity in sepsis, adding novel mass cytometry data generated using clinical samples from critically ill patients with sepsis enrolled in a randomized trial.

Our most robust finding, based on a machine learning analysis of nearly 1.5 million cytometry events, is the presence of 5 broad neutrophil clusters in patients with sepsis that can be distinguished primarily on the basis of OLFM4 and CD177 expression. We found residual heterogeneity within these clusters, particularly with regard to GLUT1, lactoferrin, and CD16 expression, among others. Signal transduction molecules including toll-like receptors and NFkB contributed very little to cluster differentiation.

Our exploratory findings are those that reflect differences between patients and time points, and are therefore based on a relatively small sample. We identified differences between patients in baseline neutrophil cluster proportions, although Cluster 1 and Cluster 3 were dominant in all but a few cases. Our results suggest that between day 1 and day 7 there is a transition from Cluster 3 to Cluster 1 neutrophils, mediated primarily by a gain in OLFM4 and CD16 expression. This effect appeared more pronounced in the placebo group than in the vitamin C group, with OLFM4 expression increasing substantially over time in the former, while decreasing slightly in the latter. Nonetheless, our more stringent linear modeling failed to show a significant interaction between temporal change and treatment arm, for either the clusters or the individual markers. This may have been due to the limited number of patients in the pairwise analysis.

Previously regarded as a homogeneous population of innate immune effector cells, neutrophils are now recognized as heterogeneous. This recognition is supported by experimental data derived from conventional flow cytometry, gradient separation, and single-cell RNA sequencing [9], which confirm multiple distinctions among neutrophil subsets. Fridlender et al. observed polarization of tumor-associated neutrophils into so-called N1 and N2 subtypes, with the former broadly interpreted as being anti-tumorigenic, and the latter more permissive of tumor growth [15]. Subsequent studies extended the N1/N2 paradigm beyond cancer, showing that similar polarization could be achieved with peripheral blood neutrophils from healthy donors incubated with either a pro-inflammatory cocktail (to produce an N1 phenotype), or one that mirrors a tumor microenvironment (for N2 polarization) [16].

Evidence of neutrophil heterogeneity in sepsis is less established, with most studies using mouse models or neutrophils from healthy controls that are activated in vitro [17–19]. Heterogeneity has been described in terms of neutrophil maturation, with aging neutrophils characterized by a gain in CXCR4 showing an increase in pro-inflammatory activation, and neutrophil extracellular trap (NET) formation [20]. Kwok et al. examined neutrophils from patients with sepsis (*n* = 26) using single-cell RNA sequencing as well as surface protein profiling to evaluate nearly 273,000 cells, linking a neutrophil subtype with a leukocyte transcriptome-defined sepsis subtype [21, 22]. Parthasarathy et al. showed that neutrophils could be divided into two subgroups based on the presence or absence of CD177 and identified differences between neutrophils from patients with sepsis and those from healthy controls. These differences were based on nine markers, which were either statistically downregulated in sepsis, including CD10, CD16, and CD86, or upregulated in sepsis, including HLA-DR, CD11b, CD80, CD184, CD63, and CD66b [23]. Mass cytometry was used in a study by Meghraoui-Kheddar et al. to identify two neutrophil subtypes that distinguished sepsis from non-infectious inflammatory states, based largely on the abundance of CD123 and PD-L1.

In distinction to these studies, our focus was on identifying differences within sepsis, rather than differences between sepsis and healthy controls. We used a different panel of neutrophil markers that were designed to evaluate the state of activation of these cells and we analyzed a large number of cytometry events that were gated as neutrophils.

Our results show that OLFM4, CD177, and GLUT1 were the most heterogeneously expressed markers in our CyTOF panel. OLFM4 is a neutrophil granule protein that has been implicated in the negative regulation of neutrophil bactericidal activity and has previously been used to define a subpopulation of neutrophils [24, 25]. Evidence for bactericidal action comes from studies in mice that show *OLFM4*^*−*^*/*^*−*^ knockouts exhibit increased intracellular killing of both *S. aureus* and *E. coli*, as well as enhanced survival in an intraperitoneal model of infection [26, 27]. In gene expression studies of patients with sepsis, increased OLFM4 expression has been associated with the development of acute respiratory distress syndrome (ARDS) [28], as well as mortality [29].

Kangelaris et al. used flow cytometry to identify OLFM4^+^ neutrophils from patients with sepsis [30]. On average, the percentage of OLFM4^+^ neutrophils was significantly higher among those who died (*n* = 24) than among those who survived (*n* = 73); however, there was extensive overlap in OLFM4 positivity between these two groups, with a mean percentage of 33.6% OLFM4^+^ cells in patients who died *vs.* 24.2% in the patients who survived. Patients with OLFM4^+^ neutrophils ≥ 37.6% were significantly older than patients with OLFM4^+^ neutrophils < 37.6%, and there was no correlation between the prevalence of OLFM4^+^ neutrophils and any of 17 inflammatory cytokines tested [30].

CD177 is a cell surface marker that has been implicated in endothelial transmigration [31], and has been shown to be overexpressed in sepsis, compared with non-sepsis patients and healthy controls [31, 32]. The expression of CD177 in human neutrophils has previously been shown to be heterogeneous, both in healthy volunteers and in patients with sepsis [31, 33, 34]. One study using microarray-based gene expression profiling found that CD177 mRNA had the highest fold-change between patients with sepsis and healthy controls [27]. In a study of ICU patients admitted with sepsis, CD177 expression was found to vary between individuals and was associated with decreased platelet interactions and reduced formation of NETs [32].

Martinez-Paz et al. examined gene expression profiles of patients with post-operative shock and found that the two most significant transcripts for differentiating mortality risk were OLFM4 (log_2_-fold 1.88) and CD177 (log_2_-fold 1.79) [35]. That study relied on transcripts from whole blood samples; therefore, the abundance of these two transcripts may not reflect changes in neutrophil subsets or clusters but indeed merely an increase in the total number of neutrophils in the blood, as suggested by an increased expression of MPO mRNA as well (log_2_-fold 0.97).

GLUT1 is a key glucose transporter on the surface of neutrophils that enables the uptake of extracellular glucose under various metabolic conditions. While neutrophils primarily rely on intracellular glycogen stores during resting states, a shift toward extracellular glucose uptake and utilization is observed in the activated state, supporting the energy demands of phagocytosis, neutrophil extracellular trap (NET) release, and the oxidative burst [36]. One recent in vitro study showed that stimulation with zymogen, TNFα, and other factors leads to a fivefold increase in GLUT1 on the cell surface within as little as 30 min. In our study, while the clusters with high OLFM4 abundance generally showed low GLUT1 abundance, some regions of cluster 3 showed high GLUT1 abundance. Conversely, small regions of clusters 1, 2, and 5 showed high GLUT1 abundance in the absence of OLFM4 expression. Discordance between the abundance of GLUT1 and some of the other markers of activation suggests this marker adds information, reflecting a more complex heterogeneity of activation states.

Consistent with prior work, we observed stark heterogeneity in the abundance of both CD177 and OLFM4 in the neutrophils of patients with severe sepsis. However, we further observed that this heterogeneity was multidimensional; we identified clusters in which CD177 and OLFM4 abundances were concordant (e.g., Cluster 3 [CD177^high^OLFM4^high^], as well as clusters 2 and 4 [CD177^low^OLFM4^low^]), but also identified Clusters with discordant expression (e.g., Cluster 1, CD177^high^OLFM4^low^, and Cluster 5, CD177^low^OLFM4^high^).

While studies of neutrophil heterogeneity have deployed several different experimental strategies, a consistent finding has been the polarization of neutrophil phenotypes along a continuum of inflammatory potential. Whether on the basis of cellular age, activation by DAMPs, or other differentiating factors, current evidence suggests the presence of neutrophil subtypes that both promote and resolve inflammatory states [9]. Our findings add to this literature by illustrating heterogeneity along additional domains, as evidenced by the differential abundance of inflammatory markers in the clusters identified. The two dominant clusters in our 5-cluster model differed largely on the basis of OLFM4 expression, again illustrating a possible polarization along a pro-inflammatory and inflammation-resolving axis.

Further supporting this concept are the exploratory analyses comparing clusters and markers between Day 1 (prior to the first dose of the study drug) and Day 7. They showed a general increase in the dominant OLFM4^high^ cluster (Cluster 3), and a small but statistically significant increase in CD16 expression, both of which may represent an ongoing inflammatory response. The analyses stratified by treatment arm showed that the transition from Cluster 1 to Cluster 3 was primarily limited to patients in the placebo arm, and that on average, those patients randomized to the vitamin C arm did not have an increase in OLFM4 expression. Vitamin C has been shown to inhibit NFkB activation in vitro [38, 39], which in turn regulates OLFM4 expression [40]. We did not, however, see a concurrent decrease in NFkB, although the dynamic range of this marker was considerably lower.

Complex neutrophil heterogeneity may offer hypotheses about differential response to various sepsis treatments. For example, eritoran, a TLR4 inhibitor, was previously shown to be ineffective in the treatment of severe sepsis, despite sound biological rationale supporting its therapeutic potential [41]. Our results reveal a narrow dynamic range of TLR4 expression across neutrophil subsets, suggesting no clear pathway to re-evaluating this treatment in any particular subgroup. Conversely, recombinant lactoferrin has also been shown to be ineffective in clinical trials [42], but unlike TLR4, it was found to have a wider range of expression across neutrophil clusters. This suggests the potential to re-visit the use of lactoferrin in sepsis, targeting a more precise subgroup of patients, or timing of administration. This sort of precision medicine approach has recently shown promise, with the ImmunoSep trial illustrating the use of immunologic subtyping to guide enrollment criteria for a study of immunomodulatory sepsis treatment [43].

Our study has a number of key strengths. Unlike prior studies, we used clinical samples from critically ill patients with sepsis enrolled in a randomized trial. With this design, we were also able to analyze samples at two different time points, enabling an evaluation of dynamic changes in neutrophil subtypes. Randomization also enables a causal inference to be made about the effect of vitamin C on the changes seen in marker and cluster abundance between the two time points. We characterized neutrophils using mass cytometry, a modality that provides additional and complementary information to existing studies of neutrophil heterogeneity. This technique allowed for the use of a comprehensive panel of markers reflecting neutrophil maturation and activation.

Some limitations bear further discussion. Due to the costs and logistical complexities of CyTOF analysis, this was done as a sub-study of the larger LOVIT trial and included only a small proportion of the overall study participants. This was compensated by the fact that the unit of analysis in our study was the neutrophil rather than the individual patient, with there being more than 1.5 million observations of the former. Nonetheless, the small patient cohort reduced statistical power, in particular for the time series analysis which required the presence of a sample on Day 7, thereby making these findings hypothesis-generating. The limited size of the cohort also makes it difficult to draw definitive conclusions about the association between neutrophil cluster proportions and clinical outcomes. Some of the patients included had only Day 1 samples; non-random missingness in the second time point has the potential to introduce bias, although there was a near equal balance of missingness due to early death and missingness due to discharge. We recruited only patients with sepsis requiring vasopressor treatment; future studies may benefit from including non-septic patients with infection, as well as healthy controls, in order to better determine if the heterogeneity we observed was specific to sepsis states. Lastly, while the parent study was multi-center in nature, all the patients recruited into this sub-study were from a single institution, which may have resulted in selection bias.

## Conclusion

We identified neutrophil subsets in blood samples from critically ill patients with sepsis, illustrating heterogeneity with potential implications for the regulation of inflammation in the innate immune response. While mass cytometry is a viable modality for studying sepsis biology, further work is needed to characterize neutrophil subtypes in larger cohorts to identify causal links between neutrophil subtypes, outcomes, and sepsis treatment responses.

## Supplementary Information

Supplementary material 1.

## Data Availability

The datasets used and/or analyzed during the current study are available from the corresponding author on reasonable request.
