# Supplementary material for: Mass cytometry reveals complex neutrophil heterogeneity in patients with severe sepsis
Source: Intensive Care Med Exp. 2026 Jun 11;14:72. doi: 10.1186/s40635-026-00924-2 (PMC13260518; doi:10.1186/s40635-026-00924-2)
Supplement: Supplementary file 1 — Supplementary material 1. [file 40635_2026_924_MOESM1_ESM.pdf]

SUPPLEMENTARY MATERIAL

Mass cytometry reveals complex neutrophil heterogeneity in patients with septic shock

Patricia D. A. Lima[1,7], Christina Yu[1], Miranda Hunt[2], Francois Lamontagne[3], Neill K.J. Adhikari[4,5], John C. Marshall[5,6], Charles C.T. Hindmarch[1,7,8], David M. Maslove*[2,7,9]

[1]Queen's Cardiopulmonary Unit, Queen's University, Kingston, Ontario, Canada
[2]Department of Critical Care Medicine, Queen's University, Kingston, Ontario, Canada
[3]Department of Medicine, Université de Sherbroooke, Sherbrooke, Québec, Canada
[4]Department of Critical Care Medicine, Sunnybrook Health Sciences Centre, Toronto, Ontario, Canada
[5]Interdepartmental Division of Critical Care Medicine, University of Toronto, Toronto, Ontario, Canada
[6]Department of Surgery, University of Toronto, Toronto, Ontario, Canada
[7]Department of Medicine, Queen's University, Kingston, Ontario, Canada
[8]Department of Biomedical and Molecular Science, Queen's University, Kingston, Ontario, Canada
[9]Kingston Health Sciences Centre, Kingston, Ontario, Canada

Supplementary Methods ………………………………………………………………………………… Page 2

Supplementary Table 1 – Antibodies and conjugates....…………………………………............... Page 6

Supplementary Table 2 – Changes in markers stratified by treatment arm……………………… Page 7

Supplementary Methods

Study patients

All patients were recruited at Kingston Health Science Center (KHSC), a tertiary academic health sciences center in Ontario, Canada. Enrollment criteria for the CyTOF sub-study were the same as for the LOVIT trial overall. Patients (≥ 18 years old) admitted to the ICU with proven or suspected infection and being treated with continuous intravenous infusions of vasopressors (norepinephrine, epinephrine, vasopressin, dopamine or phenylephrine) were included. Exclusion criteria were ICU length of stay greater than 24 hours, glucose-6-phosphate dehydrogenase deficiency, pregnancy, known allergy to vitamin C, kidney stones within the past year, expected death or withdrawal of life-sustaining treatments within 48 hours, previous intravenous vitamin C supplementation during hospitalization (unless as part of parenteral nutrition), or previous enrolment in the LOVIT study. Selection for the sub-study occurred prospectively and without knowledge of study treatment assignment. Enrollment was largely determined by the patients' willingness to participate, and recruitment during working hours when samples could be taken immediately to the CyTOF lab. For this translational study, blood was collected from patients on day 1 (D1), the day of randomization, and again on day 7 (D7) for patients still in the ICU at that time. The D1 samples were drawn prior to the first dose of study medication. Treatment arm (vitamin C vs. placebo) was not considered in the analysis since this was assigned at random, and therefore considered to have a low risk of biasing the results.

Mass Cytometry

To determine the optimal method to stabilize, fix, permeabilize, and stain the samples, we tested three different protocols in which the sequence of these steps varied. This experiment compared three methods for processing samples from three healthy individuals (ages 35 to 45; one male and two females) with no history of pathology, medical conditions, or pregnancy. These methods were:

- Method 1: fresh blood was stained with antibodies to detect surface markers -> sample was fixed and lysed (RBC) with PROT1 -> permeabilized with MeOH -> stained with antibodies to detect intracellular markers -> Cytof
- Method 2: fresh blood was stained with antibodies to detect surface markers -> sample was fixed with PROT1 -> sample was frozen (-80 °C) -> defrosted -> sample was lysed (RBC) with PROT1 -> permeabilized with MeOH -> stained with antibodies to detect intracellular markers -> Cytof
- Method 3: fresh blood was fixed with PROT1 -> sample was frozen (-80 °C) -> defrosted -> lysed with PROT1 -> sample was stained with antibodies to detect surface markers -> permeabilized with MeOH -> stained with antibodies to detect intracellular markers -> Cytof

As demonstrated in the **Supplemental Methods Figure** below, our selection of Method 3 was predicated on findings indicating that neither surface nor intracellular markers were affected by immediate fixation with PROT1. Although concerns regarding the quality of surface staining post-fixation were acknowledged, this testing confirms that no adverse effects were observed for markers such as CD3, CD16, CD15, or CD14. Furthermore, our preference for Method 3, incorporating immediate fixation, was guided by two additional objectives: 1) to minimize staining variability attributable to technical factors—given that all samples could be stained concurrently utilizing the same master mix—and 2) to reduce cellular activation during live surface staining, which immediate fixation effectively prevents, thus providing the most accurate representation of the cellular state at the time of blood collection.

For the main experiment, we collected 6 ml of blood in BD® Vacutainer Heparin tubes (Beckman, Dickson and Company, BD, USA) at the specified time points. We added 1.4 ml of the proteomic stabilizer PROT1 (Smart Tube Inc., USA) to each ml of blood, then gently mixed and incubated the samples at room temperature for 10 min as per manufacture's

recommendation. Samples were stored at 80°C until the completion of sample collection. We thawed frozen samples at 4°C, followed by red blood cell lysis using the 1x thaw-lysis buffer (Smart Tube Inc.) at the ratio of 4:1 (buffer:sample) and incubated for 10 min. Samples were centrifugated 600 g for 5 min to pellet cells. Cells were incubated with 25 ml of thaw-lysis buffer again to guarantee total lysis of red blood cells. Isolated leukocytes were washed in MaxPar cell staining buffer (Standard Bio Tools, USA) and centrifuged 800 g for 5 min, and resuspended in 1 ml of MaxPar cell staining buffer with heparin (100 U/ml; Sigma-Aldrich) for 20 min at room temperature. 270 ul of cell suspension was stained in a 30 ul of cocktail of antibodies to identified cell surface proteins (**Supplemental Table 1**) for 30 min at room temperature with gently shaking. Cells were washed twice in MaxPar cell staining buffer (800 g for 5 min), and permeabilized with cold Methanol for 20 min at 4°C. Cells were washed twice with MaxPar cell staining buffer (800 g for 5 min) and resuspended in 30 ul of Max fix perm buffer (Standard Bio Tools) containing a cocktail of antibodies to detect intracellular proteins (**Supplemental Table 1**; 30 min at room temperature), then washed with MaxPar cell staining buffer, and stained with the cell ID intercalator iridium (191-Ir and 193-Ir; Standard Bio Tools), as per manufacturer's instructions. Just before data acquisition, cells were washed in water and counted. 10^6 cells were resuspended in 1 ml of water containing 0.1X of Four Element Calibration Beads (Standard Bio Tools). All samples were processed and stained at the same time to reduce technical discrepancies, and protocol was tested and validated prior to studied samples. Data from the samples were acquired using the Helios (Standard Bio Tools), a high-performance mass cytometer from the CyTOF (time-of-flight detection) family of instruments located at the Queen's CardioPulmonary Unit (QCPU), Queen's University.

Lineage Markers (surface): CD3, CD16, CD15 and CD14

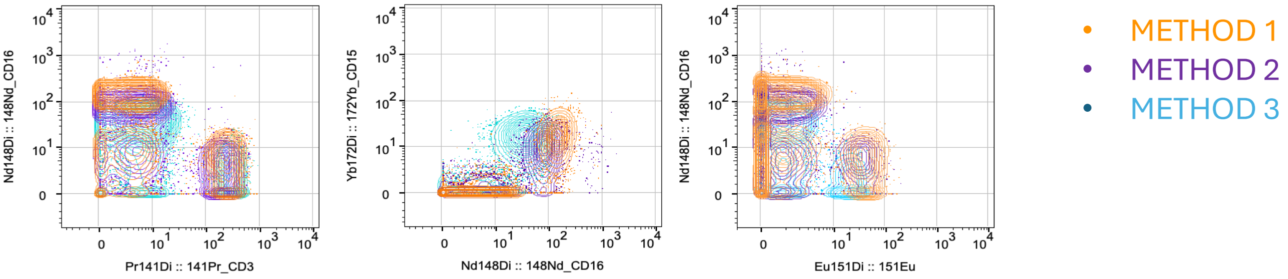

Chemokine receptor (surface): CXCR1 and CXCR2

Transcription Factors (intracellular): pNFKb and pERK

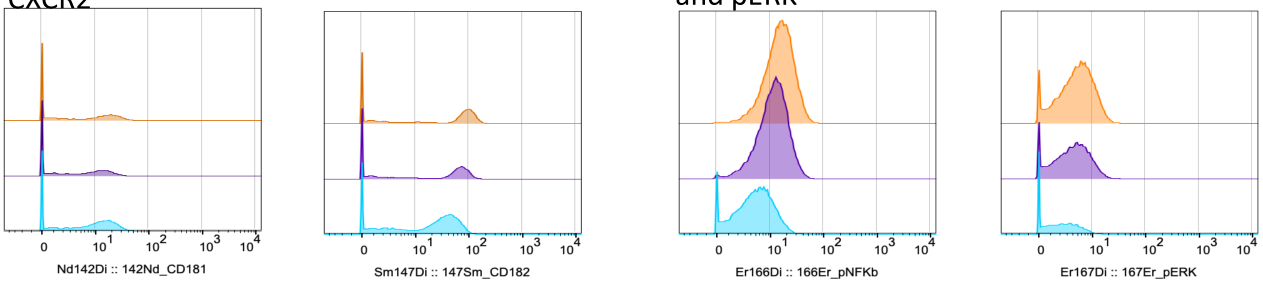

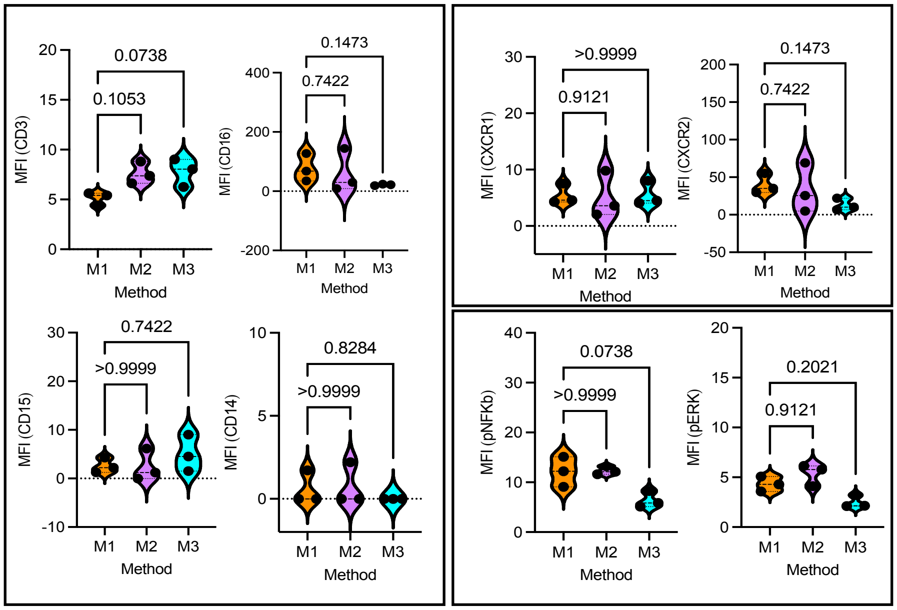

Supplemental Methods Figure: Blood samples obtained from healthy control individuals were utilized to standardize and assess the feasibility of the protocol intended for stabilizing, fixing, permeabilizing, and staining samples from sepsis patients. This evaluation involved three healthy volunteers (aged 35 to 45 years; comprising one male and two females) with no prior history of pathological conditions, health issues, or pregnancy. The plots of the different methods were merged (orange for method 1, purple for method 2, and blue for method 3) to facilitate comparative analysis. Lineage markers, which identify specific cell populations, were displayed in dot plots, demonstrating clear differentiation among cell types that was consistent across all methods. Chemokine receptors and transcription factors were shown as histograms, with observations indicating consistent staining quality regardless of the technique used. A statistical analysis utilizing the non-parametric Kruskal-Wallis test revealed no significant differences in staining intensity among the methods.

Supplemental Table 1: Antibodies' list

Conjugated Antibodies

Cat #	Metal Isotope	Target	Clone	Company
3089003B	89Y	CD45	HI30	Standard Bio Tools
3141019B	141Pr	CD3	UCHT1	Standard Bio Tools
3149021B	149Sm	CD56 (NCAM)	NCAM16.2	Standard Bio Tools
3165025B	165Ho	CD19	HIB19	Standard Bio Tools
3151009B	151Eu	CD14	M5E2	Standard Bio Tools
3148004B	148Nd	CD16	3G8	Standard Bio Tools
3144019B	144Nd	CD15 (SSEA-1)	W6D3	Standard Bio Tools
3147010B	147Sm	CXCR2	5E8/CXCR2	Standard Bio Tools
3142009B	142Nd	CXCR1	8F1/CXCR1	Standard Bio Tools
3158024B	158Gd	TLR4	HTA125	Standard Bio Tools
3176021B	176Yb	TLR2	TL2.1	Standard Bio Tools
3166006A	166Er	pNF-kB p65 [S529]	K10-895.12.50	Standard Bio Tools
3167005A	167Er	pERK 1/2 [T202/Y204]	D13.14.4E	Standard Bio Tools
3172023A	172Yb	cleaved casp 3	5A1E	Standard Bio Tools

Antibodies that were conjugated with metals*

Cat #	Metal Isotope	Target	Clone	Company
ab95681	163Dy	GM-CSFR	4H1	Abcam
MCA2045	153Eu	CD177	MEM 166	Bio-Rad
ab115730	164Dy	Glut1	EPR3915	Abcam
ab136180	162Dy	Glut3	-	Abcam
MCA1757	175Lu	MPO	2C7	Bio-Rad
ab135710	160Gd	Lactoferrin	-	Abcam
ab85046	155Gd	OLFM4	-	Abcam
ab210073	159Tb	HIF-1a	EP1215Y	Abcam
ab23477	174Yb	Lipocalin-2	5G5	Abcam
ab236878	152Sm	SVCT1	-	Abcam
229802	170Er	SVCT2	-	Abcam

Unconjugated antibodies were conjugated using the Maxpar antibody labeling kit (**Maxpar® X8 Multimetal Labeling Kit—40 Rxn PN#201300;** Standard Bio Tools**),** and primary antibodies passed the requirements for conjugation. Working concentration was tested for individual

Supplemental Table 2. Changes in individual markers stratified by treatment arm

marker	D1 mean	D7 mean	% change	p-value	adjusted p-value
Placebo					
MPO	0.983	0.936	-4.764	0.016	0.182
SVCT1	0.478	0.574	20.072	0.030	0.182
pERK	0.169	0.141	-16.670	0.040	0.182
OLFM4	0.470	0.643	36.737	0.049	0.182
CXCR2	0.155	0.284	83.501	0.054	0.182
NFKB	0.096	0.081	-16.188	0.060	0.182
CD16	0.615	0.789	28.451	0.081	0.182
TLR2	0.147	0.128	-12.875	0.085	0.182
CD15	0.422	0.505	19.794	0.087	0.182
HIF-1a	0.150	0.130	-12.852	0.091	0.182
cCasp3	0.047	0.043	-10.332	0.102	0.186
CD177	0.977	1.057	8.114	0.169	0.281
CXCR1	0.342	0.401	17.436	0.210	0.319
GLUT-1	0.190	0.304	59.375	0.223	0.319
SVCT2	0.236	0.215	-8.824	0.285	0.380
LACTO	0.640	0.685	7.083	0.323	0.403
GM-CSFr	0.119	0.127	6.314	0.561	0.660
TLR4	0.146	0.140	-3.587	0.672	0.746
GLUT-3	0.235	0.228	-3.155	0.725	0.746
lipocalin-2	1.154	1.159	0.441	0.746	0.746
Vitamin C					
CXCR1	0.249	0.350	40.639	0.023	0.222
MPO	0.982	0.955	-2.798	0.023	0.222
SVCT1	0.410	0.525	27.905	0.033	0.222
lipocalin-2	1.101	1.140	3.567	0.051	0.253
CD16	0.608	0.807	32.826	0.075	0.298
CXCR2	0.105	0.267	153.772	0.110	0.363
TLR2	0.163	0.126	-22.939	0.127	0.363
CD15	0.473	0.529	12.046	0.209	0.489
GLUT-3	0.224	0.182	-18.908	0.220	0.489
NFKB	0.092	0.074	-18.863	0.310	0.621
GLUT-1	0.282	0.352	24.796	0.358	0.651
HIF-1a	0.137	0.122	-11.287	0.448	0.746

pERK	0.152	0.142	-6.132	0.668	0.994
GM-CSFr	0.112	0.120	6.386	0.712	0.994
LACTO	0.600	0.617	2.796	0.748	0.994
OLFM4	0.661	0.643	-2.732	0.841	0.994
cCasp3	0.050	0.049	-2.288	0.863	0.994
SVCT2	0.214	0.216	1.079	0.896	0.994
TLR4	0.126	0.127	0.294	0.984	0.994
CD177	1.045	1.045	-0.037	0.994	0.994

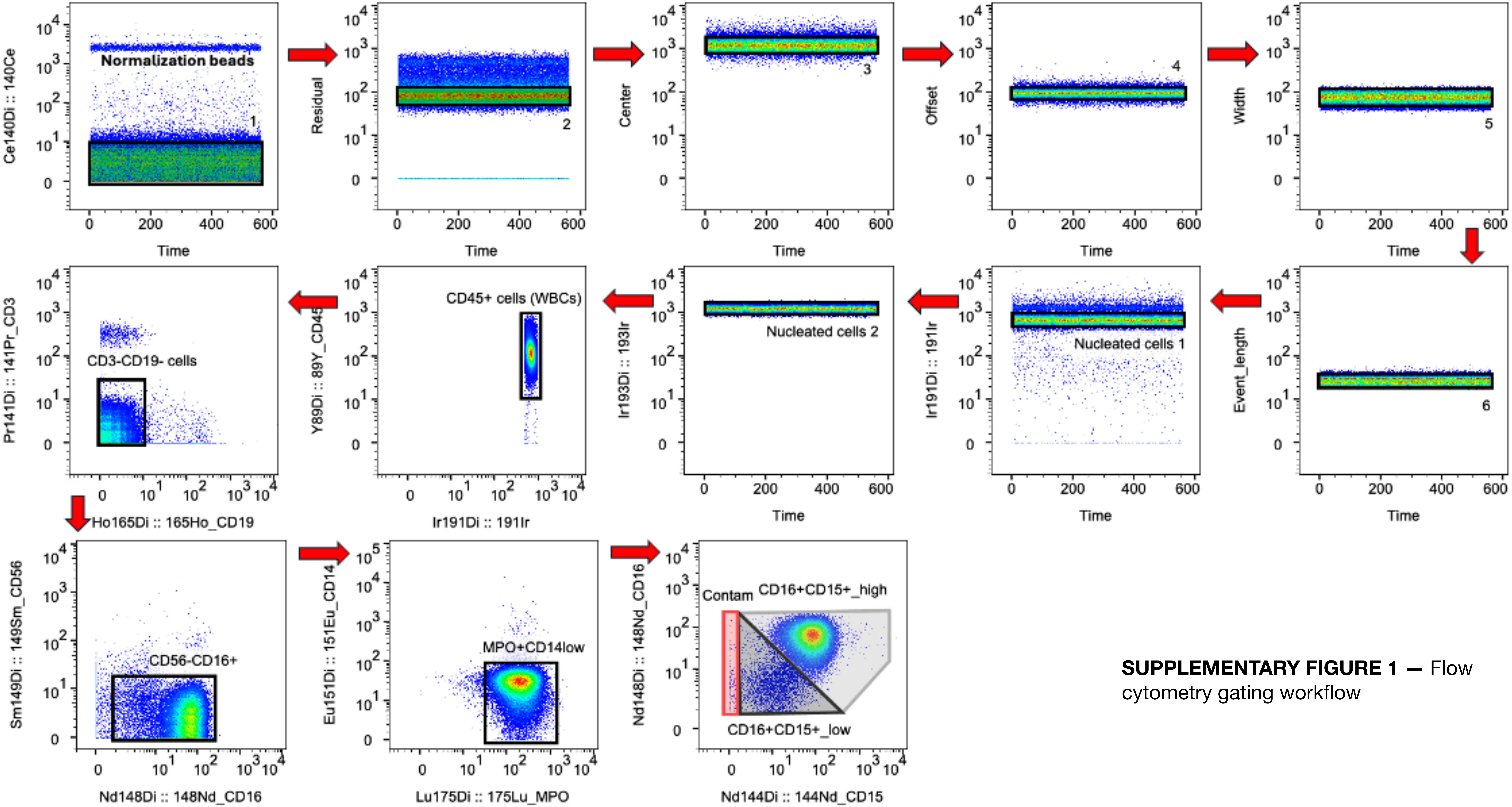

SUPPLEMENTARY FIGURE 1 — Flow cytometry gating workflow

SUPPLEMENTARY FIGURE 2 — Non-redundancy scores for each neutrophil marker in the CyTOF panel. Each dot represents a sample in the experiment. Colour corresponds to specific sample (eg. “Patient 1, Day 1”). Markers with higher NRS account for a greater proportion of variability in the dataset.

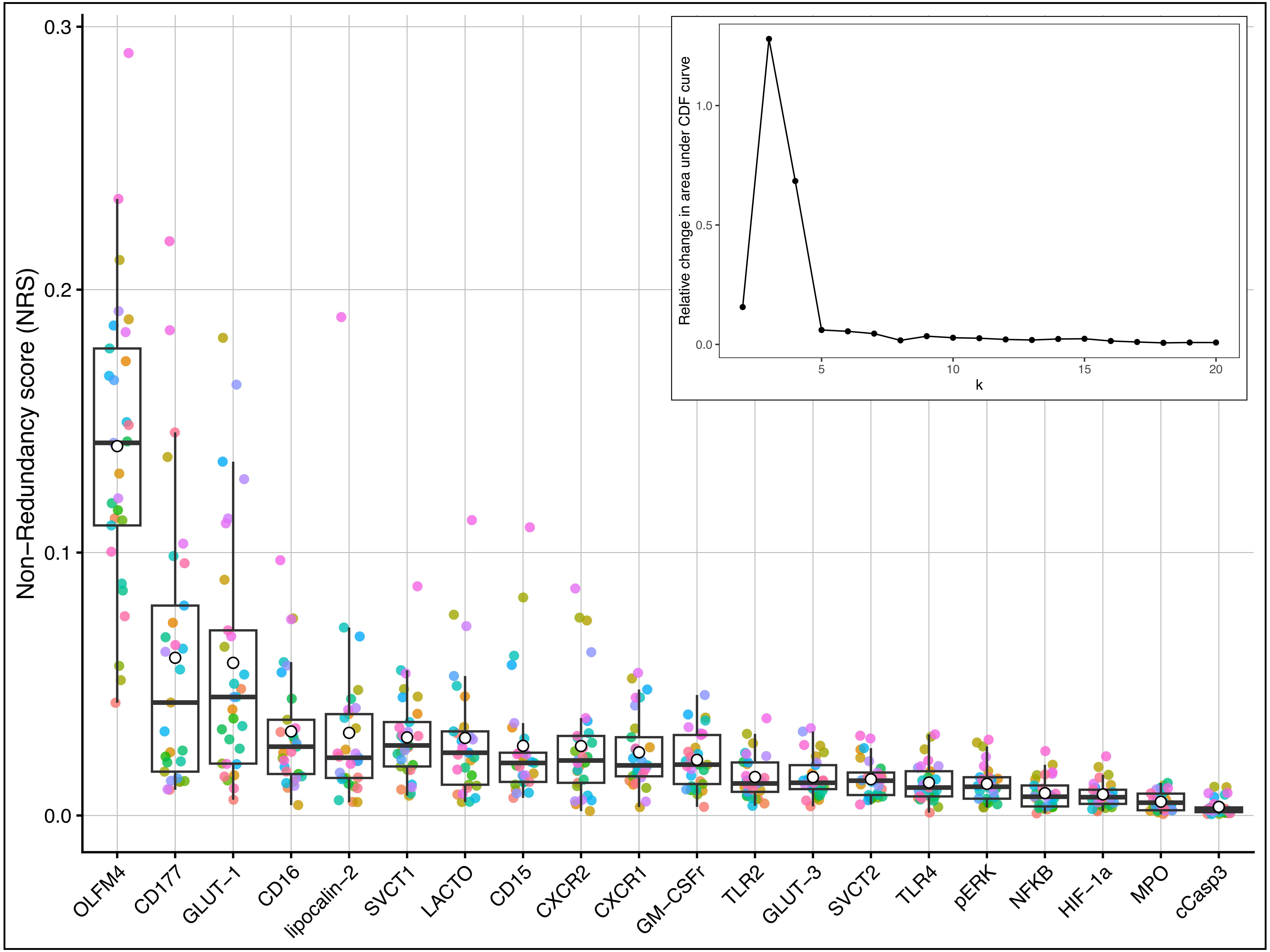

SUPPLEMENTAL FIGURE 3 — t-SNE plots for each of the markers in the CyTOF experiment.

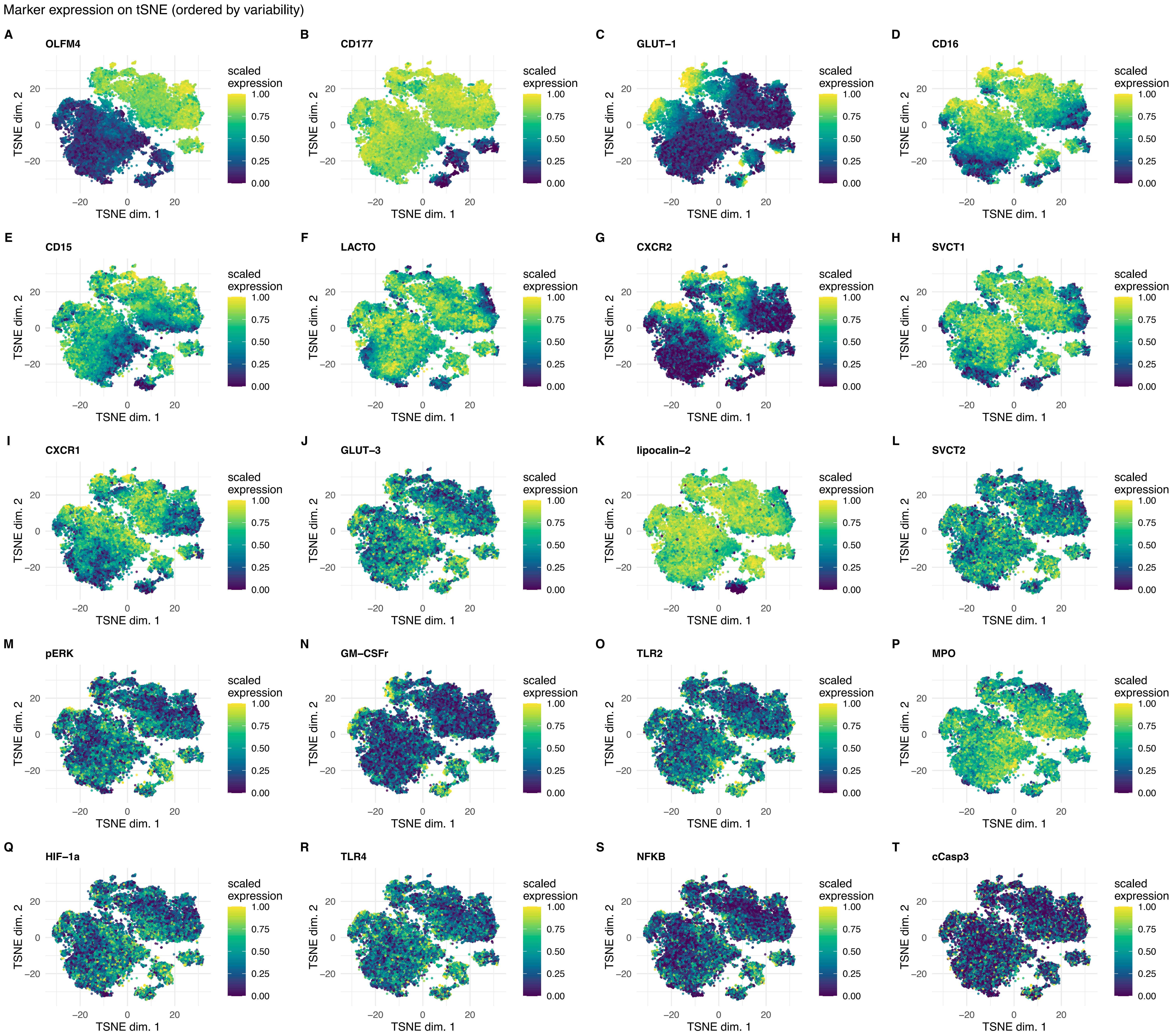

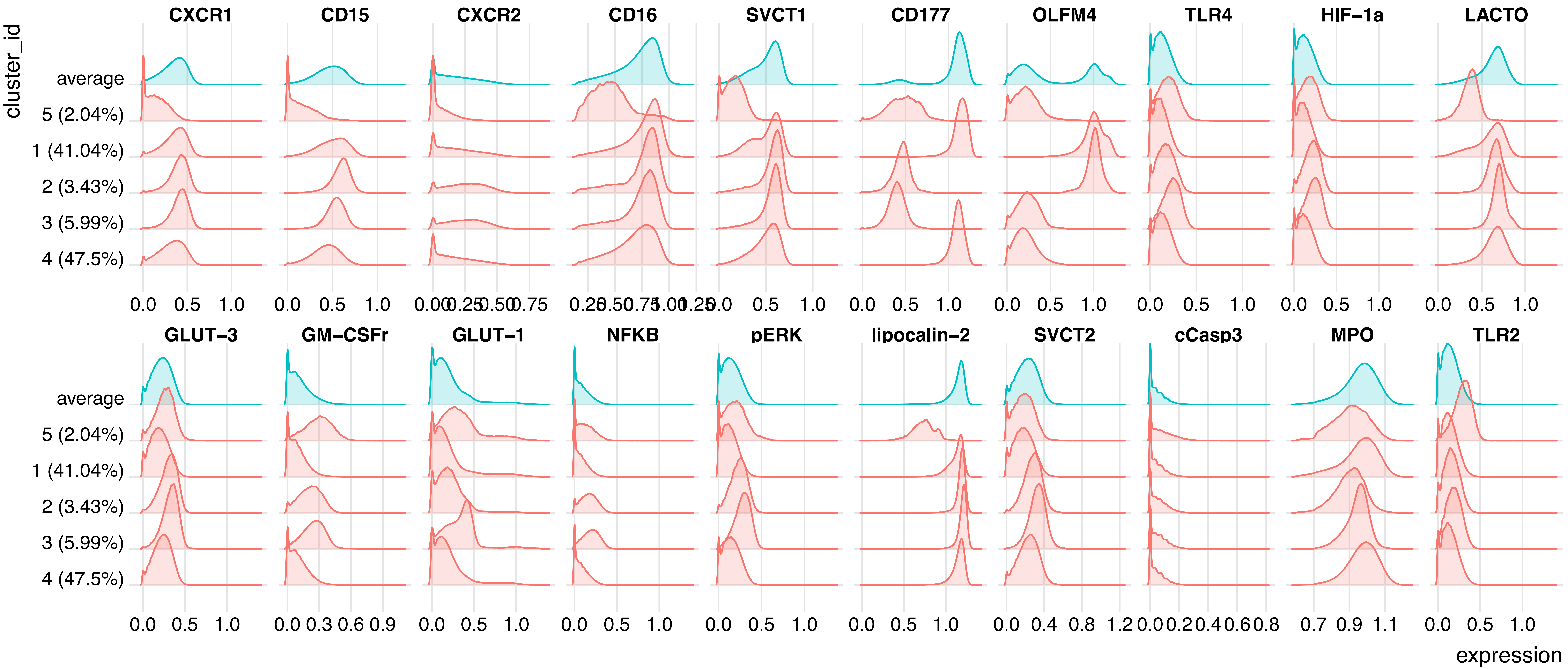

SUPPLEMENTAL FIGURE 4 — Density plots for marker intensities by cluster.

SUPPLEMENTAL FIGURE 5 — Changes in marker expression from Day 1 to Day 7, stratified by treatment arm. Orange dots (labeled “AA” for ascorbic acid) are for the vitamin C group. Black dots (labeled “Plac”) are for the placebo group.

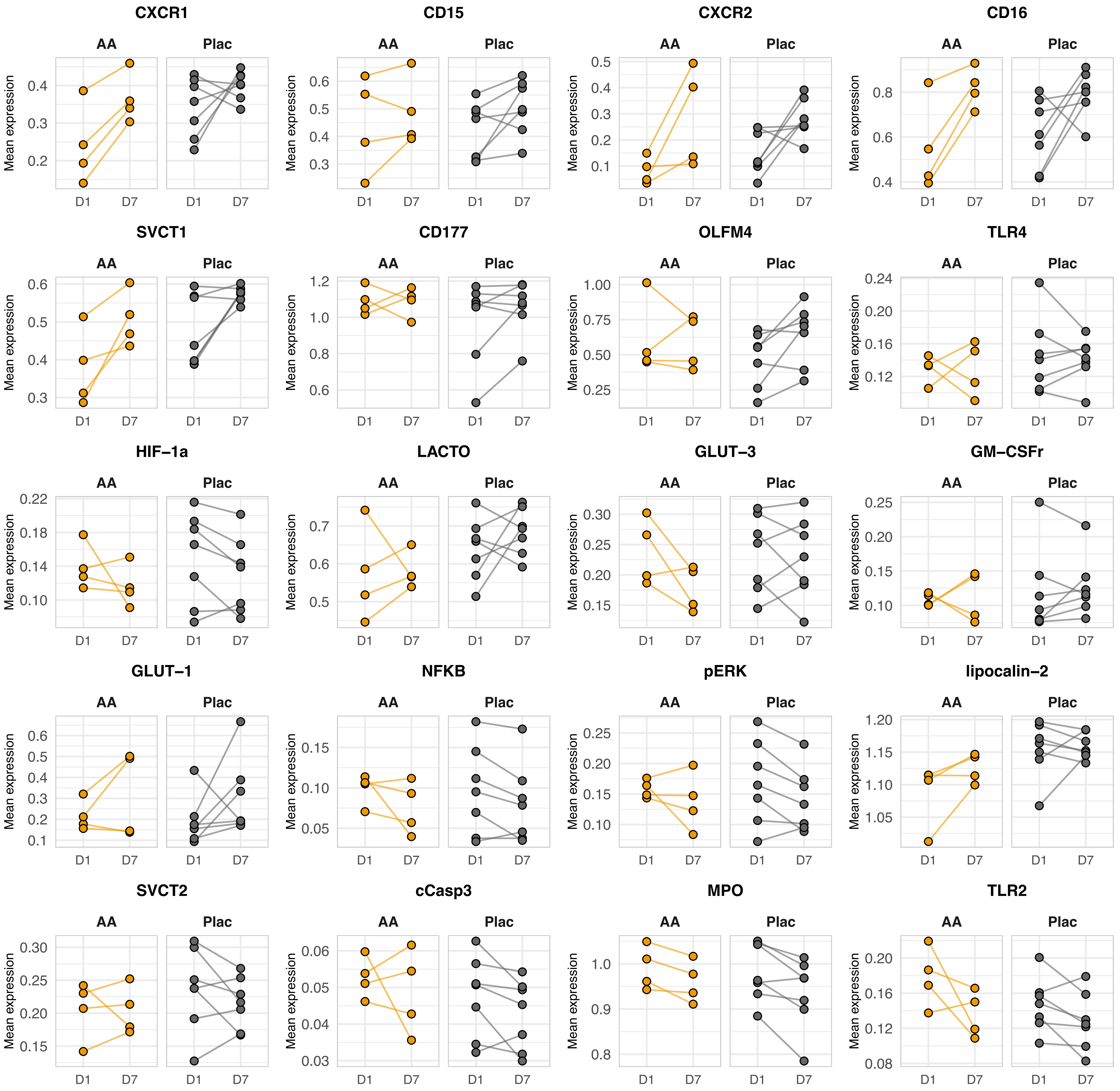

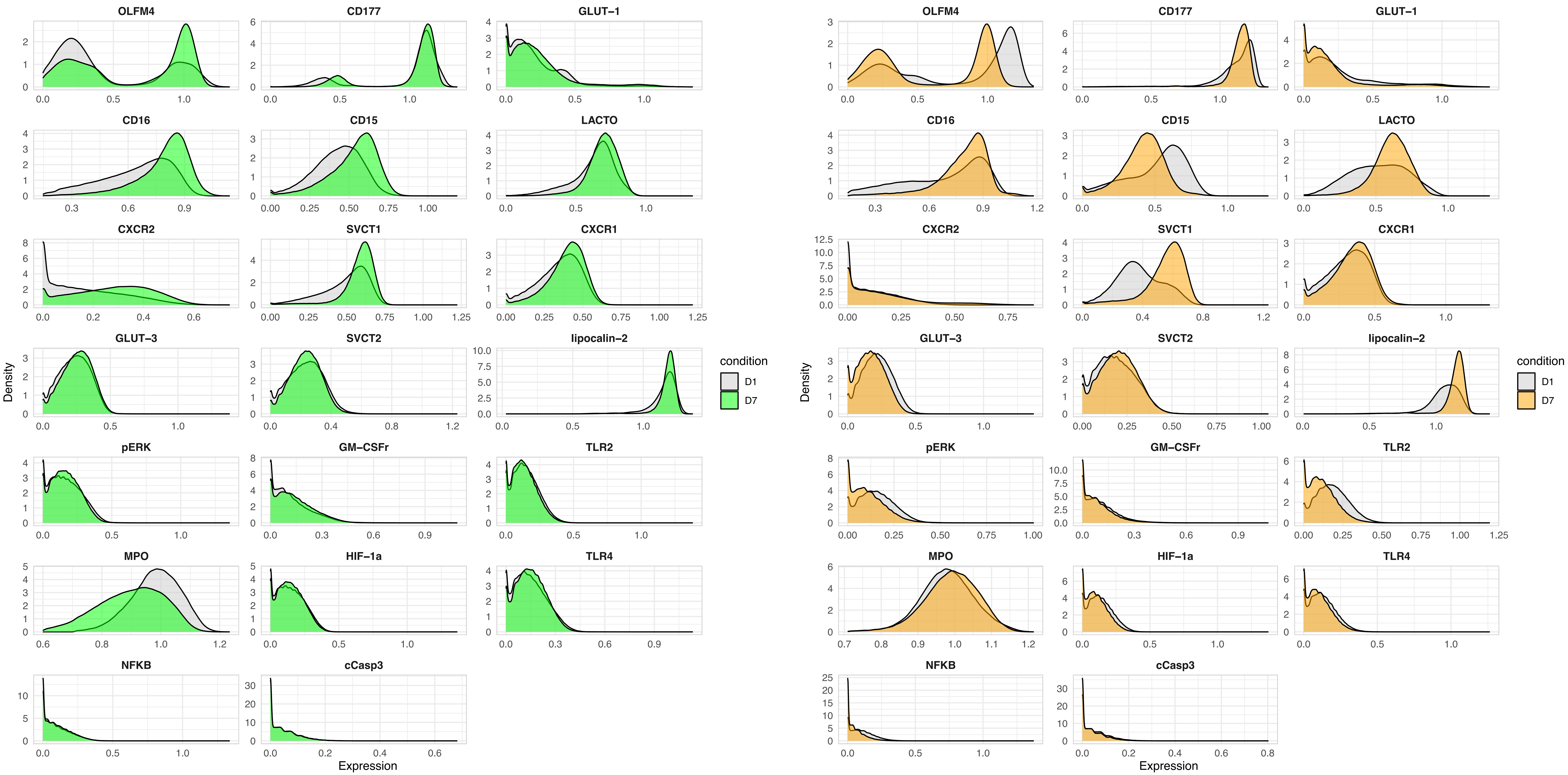

SUPPLEMENTAL FIGURE 6 — Density plots for each of the markers in the experiment stratified by treatment arm. The plots on the left are placebo arm values (grey for Day 1, green for Day 7). The plots on the right are vitamin C arm values (grey for Day 1, orange for Day 7).
